# Independent correlates of vancomycin steady-state trough concentration among patients undergoing combined CABG and valve surgery: observational findings for intra-aortic balloon pump support

**DOI:** 10.3389/fphar.2026.1888382

**Published:** 2026-10-12

**Authors:** Bei-bei Shao, Tao Feng, Ji-cheng Jiang, Wen-lu Xing, Ming Ni, Lu-lu Li, Chao Liu, Shu-ping Meng, Yong-cheng Ma, Ai-feng Wang

**Affiliations:** 1 Henan Key Laboratory of Individualized Drug Therapy for Cardiovascular Diseases, Department of Pharmacy, Fuwai Central China Cardiovascular Hospital, Central China Fuwai Hospital of Zhengzhou University, Zhengzhou, China; 2 Department of Cardiac Surgery Intensive Care Unit, Fuwai Central China Cardiovascular Hospital, Central China Fuwai Hospital of Zhengzhou University, Zhengzhou, China; 3 Department of Cardiology, Big Data Center for Cardiovascular Disease, Fuwai Central China Cardiovascular Hospital, Central China Fuwai Hospital of Zhengzhou University, Zhengzhou, China

**Keywords:** CABG, cardiac surgery, IABP, individualized medication, steady-state trough concentration, TDM, vancomycin

## Abstract

**Background:**

Patients undergoing coronary artery bypass grafting (CABG) combined with valve surgery represent a high-risk surgical population with substantial perioperative hemodynamic disturbances and highly variable organ function, leading to high variability in vancomycin pharmacokinetics. Conventional vancomycin dosing solely on renal function frequently fails to achieve target trough concentrations in this complex subgroup. This study aimed to identify independent correlates c of vancomycin steady-state trough concentrations (C_ss-min_), and to report observational findings for intra-aortic balloon pump (IABP) support, to inform individualized therapeutic drug monitoring.

**Methods:**

A single-center retrospective observational study enrolled adult patients undergoing combined CABG and valve surgery from January 2018 to October 2024. Clinical data and vancomycin TDM records were extracted from electronic medical records. Vancomycin C_ss-min_ was detected by enzyme-multiplied immunoassay technique. Generalized Estimating Equations (GEE) accounting for within-patient repeated TDM measurements were used to identify clinical correlates of C_ss-min_. IABP support was pre-specified and force-entered into the multivariable model based on clinical observations and pathophysiological reasoning, independent of univariate screening results. We performed a sensitivity analysis for patients with at least one TDM within postoperative day 7, prespecified interaction tests for effect modification, and binary logistic-GEE for the supratherapeutic endpoint (C_ss-min_ > 20 mg/L). A two-tailed P < 0.05 was set as statistical significance.

**Results:**

A total of 283 eligible patients with 473 valid C_ss-min_ measurements were included. Multivariate GEE demonstrated that categorical vancomycin dosing regimen, age, ventilator dependence ≥96 h, continuous renal replacement therapy (CRRT), IABP support, serum creatinine (Scr), and serum urea nitrogen (SUN) were positively associated with higher geometric-mean C_ss-min_. Associations remained largely consistent in the sensitivity-analysis subgroup of patients with at least one early-phase TDM within postoperative day 7. A significant CRRT-by-postoperative-time interaction was detected, indicating that the influence of CRRT on vancomycin exposure varied across different postoperative phases. Of note, the association signal linking IABP to supratherapeutic trough concentrations (C_ss-min_>20 mg/L) was modest and requires cautious interpretation.

**Conclusion:**

Multiple clinical factors jointly affect vancomycin steady-state trough concentrations in patients undergoing combined CABG and valve surgery, including dosing regimen, age, renal biomarkers, and critical-care supportive interventions such as IABP, CRRT and prolonged mechanical ventilation. IABP support represents an intriguing observational signal; this finding remains hypothesis-generating and requires prospective external validation before it can guide formal dose-adjustment strategies. Individualized vancomycin management for this population should comprehensively integrate hemodynamic support measures and laboratory indices rather than depend merely on renal-function markers. This study focuses on clinically accessible trough concentration monitoring rather than constructing complex pharmacokinetic models, which is more in line with real-world clinical practice.

## Introduction

With the rising incidence of cardiovascular diseases and advancements in cardiac surgical techniques, coronary artery bypass grafting (CABG) combined with valve surgery has become a common yet complex and high-risk surgical procedure accompanied by frequent postoperative complications (Zhu et al., 2026). As reported, the overall morbidity rate of postoperative complications after cardiac surgery was 54.2%; Compared with single CABG or valve surgery, combined surgery carries a higher risk of postoperative infection and other adverse events (Zubkov et al., 2025; Jain et al., 2025). Consequently, vancomycin is frequently used as a key agent for both perioperative prophylaxis and the treatment of Gram-positive infections in cardiac surgery settings, particularly in those with a high prevalence of methicillin-resistant *Staphylococcus* species (Tsai et al., 2025; Kachroo et al., 2006; Rybak et al., 2020).

Vancomycin undergoes minimal metabolism *in vivo* and is almost entirely eliminated unchanged via renal excretion (Altowayan et al., 2023; Cantu et al., 1994; Martin et al., 2010; He et al., 2021). Such elimination characteristics results in substantial interindividual variations in drug exposure, particularly in postoperative patients with fluctuating hemodynamics and renal function. As recommended, the efficacy and toxicity of vancomycin can be predicted by the 24-h area under the concentration-time curve to minimum inhibitory concentration ratio (AUC_24_/MIC) and the steady-state trough concentration (Martin et al., 2010; Liu et al., 2024; Reuter et al., 2022; He et al., 2020). An AUC_24_/MIC range of 400–600 is generally recommended to guarantee therapeutic outcomes against severe methicillin-resistant *Staphylococcus aureus* infections (Rybak et al., 2020). Considering clinical cost and operability, steady-state trough concentration ranging from 10 to 20 mg/L is adopted as the practical therapeutic target to balance efficacy and toxicity (Liu et al., 2024; Reuter et al., 2022; He et al., 2020). Nevertheless, elevated vancomycin exposure still raises nephrotoxicity risks, which markedly increases when AUC_24_ exceeds 650 mg·h/L or trough concentration goes above 15 mg/L (Rybak et al., 2020; Reuter et al., 2022; He et al., 2020; Kim et al., 2022).

Routine vancomycin therapeutic drug monitoring (TDM) is suggested for patients with unstable renal function, renal replacement therapy and moderate to severe heart failure (He et al., 2020). Initial empirical dosage adjustment based on creatinine clearance calculated via the Cockcroft-Gault formula is preferred for renally impaired patients, and dose re-titration guided by trough concentration within 10–20 mg/L serves as a feasible clinical strategy (Reuter et al., 2022; He et al., 2020).

Achieving these targets is challenging in patients post-CABG combined with valve surgery, due to dynamic renal function changes after cardiopulmonary bypass and the need to balance efficacy with emerging concerns such as vancomycin-resistant *Enterococcus* colonization (Kachroo et al., 2006). In clinical practice, dosage formulated merely according to Crcl often results in trough concentrations exceeding 20 mg/L, and this iatrogenic vancomycin overdose may exacerbate the risk of nephrotoxicity, which is already a significant concern in this vulnerable population (Rybak et al., 2020; Liu et al., 2024).

The postoperative course is often complicated by conditions such as low cardiac output syndrome, acute kidney injury (AKI), and multi-organ dysfunction (Sakhaei et al., 2023; Duncan et al., 2022; Beaver et al., 2024). These pathophysiological states can profoundly alter vancomycin pharmacokinetics by affecting its volume of distribution and renal clearance. Optimizing vancomycin efficacy while minimizing adverse reactions remains a tough clinical challenge in this complex subgroup. This retrospective study was therefore conducted to explore independent Correlates of vancomycin steady-state trough concentrations in patients undergoing combined CABG and valve surgery, aiming to provide evidence for optimized individualized medication strategies.

## Materials and methods

### Patients

This retrospective observational study was carried out between January 2018 and October 2024 in the department of Cardiovascular Surgery of a tertiary care university hospital with 1,140 beds and more than 5,000 annual cardiac surgical procedures. Patients who met the following criteria were enrolled in the study: (1) received vancomycin for postoperative antimicrobial prophylaxis or infection treatment, (2) underwent postoperative monitoring of serum vancomycin steady-state trough concentration (C_ss-min_), (3) underwent CABG combined with concomitant valve surgery, including surgical valvuloplasty and valve replacement, and (4) age ≥18 years. Patients or TDM records were excluded if sampling failed to meet steady-state trough criteria, if the concentration was obtained preoperatively, or if records involved temporary drug discontinuation or non-standard vancomycin administration.

### Study design

Steady-state vancomycin concentrations are generally achieved after 4 to 5 consecutive doses, and the time to steady-state is prolonged in patients with impaired renal function (He et al., 2020). All patients in the present study received initial vancomycin regimens determined collaboratively by attending physicians and clinical pharmacists based on individual creatinine clearance. The most frequently used initial empirical dosing regimens were 1 g every 12 h, 0.5 g every 8 h, or 0.5 g every 12 h, diluted with 0.9% normal saline or 5% glucose solution and administered via intravenous infusion over 1–2 h. The timing of therapeutic drug monitoring (TDM) was individualized according to the vancomycin dose and dynamic renal function. Trough blood samples were uniformly collected within 30 min before the next scheduled vancomycin dose by specialized nurses to ensure standardized sampling quality.

For patients with unstable perioperative renal function, trough concentration-based dosing adjustment was preferred, whereas AUC-based monitoring was not routinely applicable in this critically ill surgical population (He et al., 2020). Given the fluctuating hemodynamics and renal function in patients undergoing CABG combined with valve surgery, steady-state trough concentration monitoring was adopted as a practical and feasible TDM strategy, with a target therapeutic range of 10–20 mg/L.

For regression analyses, all originally observed nine dosing regimens were merged into five pharmacologically-homogeneous categories: Group 1 (low-dose: 0.25 g q12h, 0.5 g qd, 0.5 g q12h), Group 2 (0.5 g q8h), Group 3 (0.5 g q6h), Group 4 (high-dose: 1 g q12h, 1 g q8h), and Group 5 (non-standard administration/temporary drug discontinuation). Group 5 records were excluded from all regimen-related regression models; Groups 1–4 were entered as a categorical covariate, with Group 1 as reference. All demographic, surgical, therapeutic, and laboratory data were retrospectively extracted from the hospital electronic medical record database.

Laboratory variables, including C-reactive protein (CRP), procalcitonin (PCT), serum creatinine (Scr), serum urea nitrogen (SUN), serum total bilirubin (STB), serum albumin (SAlb), and N-terminal pro-B-type natriuretic peptide (NT-proBNP) were collected within 72 h prior to each vancomycin TDM sampling. The study protocol complied with the Declaration of Helsinki and was approved by the Ethics Committee for Scientific Research of Fuwai Central China Cardiovascular Hospital ([2023]80).

### Vancomycin concentration assay

Vancomycin plasma concentrations were determined using the Siemens Viva-ProE automated biochemical analyzer and the EMIT® 2000 Vancomycin Assay (Siemens Healthineers, Erlangen, Germany), following the manufacturer’s instructions (Healthin et al., 2024). The measuring range was 2–50 mg/L.

### Statistical analysis

The Shapiro-Wilk test was used to assess the normality of continuous data. Continuous variables were presented as median with interquartile range, and categorical variables as frequencies and percentages. Between-group comparisons of continuous variables were performed using Kruskal-Wallis test, Mann-Whitney-U, χ^2^ or Fisher’s exact tests as appropriate. All statistical tests were two-sided, with P < 0.05 considered statistically significant. Of 473 steady-state TDM records, 13 records with non-standard dosing or temporary drug discontinuation were excluded, yielding the primary analysis dataset of 460 measurements.

Generalized Estimating Equations (GEE) with an exchangeable working correlation structure were applied to account for repeated TDM measurements within individual patients. The dependent variable was log-transformed steady-state trough concentration (log C_ss-min_). Variables significant in univariate GEE together with IABP (clinical observations and pathophysiological reasoning), body weight (vancomycin dose was not weight-adjusted), and PCT (significant in between-group comparison) were included in the multivariable GEE model. Model fit was assessed using the quasi-likelihood under the independence model criterion (QICC).

Pre-specified sensitivity analyses included: (1) GEE restricted to 217 patients with ≥1 TDM within postoperative day 7, incorporating predictor-by-time interaction terms; (2) testing for dosing-regimen-by-organ-support interactions in the full dataset; (3) logistic-GEE for binary C_ss-min_ (cut-off = 20 mg/L); (4) comparison between early-phase (≤7-day) and late-phase (>7-day) TDM measurements using linear mixed models for continuous outcomes; patient-level baseline comparisons between 217 patients with ≥1 early-phase TDM and 66 patients with only late-phase TDM were performed with Mann-Whitney-U, χ^2^ or Fisher’s exact tests.

ROC-based discriminative performance was calculated at the TDM-measurement level without adjusting for intra-patient correlation. All statistical analyses were conducted using SPSS 23.0 software (IBM Corporation, Armonk, NY, USA).

## Results

### Study population and vancomycin concentrations

According to the inclusion and exclusion criteria, 473 steady-state trough concentration (C_ss-min_) from 283 patients who underwent CABG combined with concomitant valve surgery were initially enrolled (Figure 1). Baseline characteristics of the 283 enrolled patients are summarized in Table 1. After excluding 13 non-standard or temporary-discontinuation records, the primary analysis dataset contained 460 TDM measurements. Among the initial TDM records, C_ss-min_ was <10 mg/L in 54 measurements, 10–20 mg/L in 219 measurements, and >20 mg/L in 200 measurements (Supplementary Figure 1).

**FIGURE 1:**
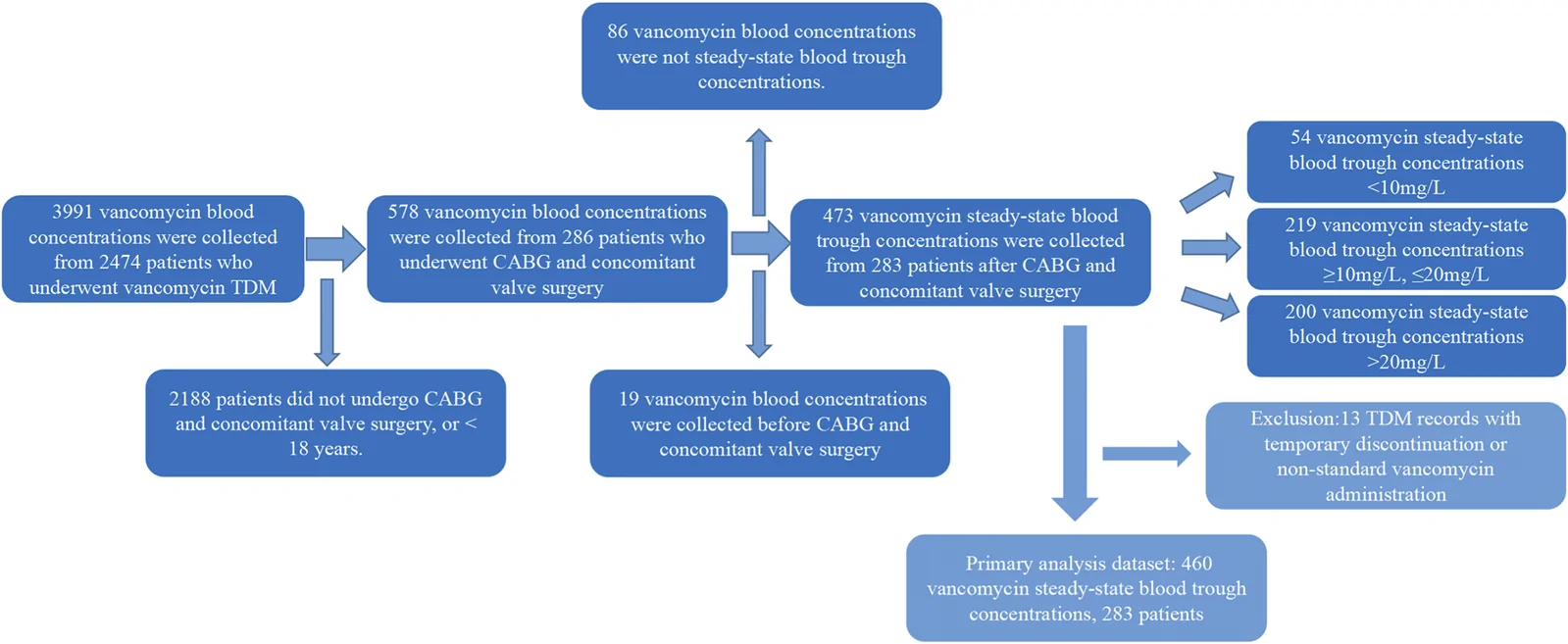
. Flow chart of the study. 3991 vancomycin TDM measurements were collected from 2474 patients. After excluding 2188 patients who did not receive combined CABG and valve surgery or were younger than 18 years, 578 measurements from 286 patients remained. Nineteen preoperative samples and 86 non-steady-state troughs were excluded, yielding 473 steady-state trough measurements from 283 patients. Thirteen records with temporary discontinuation or non-standard dosing were further excluded for the primary analysis dataset (460 measurements). Trough concentration stratification was performed descriptively on the 473 steady-state records. The exploratory observation-level subgroup GEE analysis included 261 TDM measurements within postoperative day 7. Some patients had both early and late TDM records; the remaining 212 late measurements originated from 132 patients. For sensitivity analysis, patients were categorized as having ≥1 early TDM (n=217) or only late TDM (n=66), with baseline characteristics shown in Supplementary Table S10.

**TABLE 1:** Baseline characteristics of the 283 enrolled patients.

Parameters	All patients
Patients	283
Age, years, median (IQR)	62 (14)
Male gender, n (%)	205 (72.4%)
Weight, kg, median (IQR)	68.5 (15.85)
Preoperative LVEF, median (IQR)	56% (16%)
Operative duration, h, median (IQR)	7.75 (2.75)
Ventilator dependence ≧96h, n (%)	105 (37.1%)
CRRT, n (%)	28 (9.9%)
IABP, n (%)	41 (14.5%)
ECMO, n (%)	8 (2.8%)

Footnote: Continuous variables are presented as median (interquartile range, IQR); categorical variables are expressed as number (percentage).

Baseline and treatment characteristics of TDM measurements stratified by trough-concentration subgroups are summarized in Table 2. Significant intergroup differences were observed for gender, age, ventilator dependence ≥96 h, vancomycin dosing regimen, procalcitonin (PCT), serum creatinine (Scr), serum urea nitrogen (SUN), and NT-proBNP.

**TABLE 2:** Baseline and treatment characteristics of TDM measurements stratified by vancomycin steady-state trough concentration (C_ss-min_).

C_ss-min_ (mg/L)	Total	<10 mg/L	10–20 mg/L	>20 mg/L	p-value
N	473	54	219	200	​
C_ss-min_, mg/L, median (IQR)	19.0 (11.1)	8.1 (2.6)	15.5 (5.0)	25.45 (10.0)	<0.001
Time of TDM, d, median (IQR)	7 (8)	7 (8)	7 (8)	7 (8)	0.784
Male gender, n (%)	346 (73.2%)	46 (85.2%)	165 (75.3%)	135 (67.5%)	0.004
Age, years, median (IQR)	61 (15)	56 (20)	61 (15.5)	63 (12.5)	<0.001
Weight, kg, median (IQR)	68.7 (17.3)	71.5 (19)	69.2 (17.1)	68 (16)	0.458
Preoperative LVEF, %, median (IQR)	57 (15)	57 (15)	57 (16)	55 (14)	0.327
Operative duration, h, median (IQR)	7.83 (2.91)	7.75 (2.58)	7.58 (2.66)	8.25 (2.94)	0.102
Ventilator dependence ≧96 h, n (%)	202 (42.7%)	14 (25.9%)	85 (38.8%)	103 (51.5%)	0.001
CRRT, n (%)	50 (10.6%)	4 (7.4%)	18 (8.2%)	28 (14%)	0.114
IABP, n (%)	82 (17.3%)	7 (13%)	34 (15.5%)	41 (20.5%)	0.27
ECMO, n (%)	19 (4.0%)	2 (3.7%)	9 (4.1%)	8 (4.0%)	0.991
Vancomycin dosing regimen, n (%)	​	​	​	​	0.026
Group 1: Low-dose regimen (0.25 g q12h, 0.5 g qd, 0.5 g q12h)	134 (28.3%)	17 (31.5%)	70 (32.0%)	47 (23.5%)	​
Group 2: 0.5 g q8h	154 (32.6%)	20 (37.0%)	71 (32.4%)	63 (31.5%)	​
Group 3: 0.5 g q6h	65 (13.7%)	4 (7.4%)	34 (15.5%)	27 (13.5%)	​
Group 4: High-dose regimen (1 g q12h, 1 g q8h)	107 (22.6%)	9 (16.7%)	39 (17.8%)	59 (29.5%)	​
Group 5: Non-standard regimen/temporary drug discontinuation	13 (2.8%)	4 (7.4%)	5 (2.3%)	4 (2.0%)	​
CRP, mg/L, median (IQR)	94.80 (131.64)	92.52 (123.24)	85.97 (142.96)	103.83 (129.275)	0.289
PCT, ng/mL, median (IQR)	1.78 (4.053)	0.820 (1.972)	1.510 (3.822)	2.370 (5.471)	<0.001
Scr, μmol/L, median (IQR)	98.0 (61.25)	74.5 (32)	88 (44)	127 (76)	<0.001
SUN,mmol/L, median (IQR)	14.05 (11.85)	9.05 (5.70)	11.90 (8.70)	19.00 (15.75)	<0.001
STB, μmol/L, median (IQR)	21.60 (28.15)	20.25 (21.40)	19.80 (25.00)	24.00 (35.45)	0.064
SAlb, g/L, median (IQR)	44.40 (6.60)	44.65 (5.80)	44.60 (6.55)	44.30 (6.70)	0.984
NT-proBNP, ng/L, median (IQR)	2,510.0 (3,391.0)	1727.5 (2,337.9)	2,116.0 (2,658.0)	3,240.0 (3,943.5)	<0.001

Footnote: Data are presented as median (interquartile range, IQR) for continuous variables and number (percentage) for categorical variables. Continuous variables were compared using the Kruskal–Wallis test and categorical variables using the χ^2^ test or Fisher’s exact test, as appropriate. Vancomycin dosing regimens were categorized into five groups. Group 5 (n = 13) was excluded from the multivariable GEE, analysis, which included Groups 1–4, to avoid unstable model estimation. All P-values are descriptive only and do not account for repeated TDM, measurements within individual patients.

A total of 217 patients had at least one TDM performed within postoperative day 7, contributing 261 early-phase TDM measurements prior to exclusion of non-standard regimens. For the subgroup of 217 patients with at least one early-phase TDM (375 total TDM measurements including both early- and late-phase records from these individuals), comparable inter-group difference patterns across trough-concentration strata were observed (Supplementary Table S3).

### Univariate analysis

Given the non-normal distribution of C_ss-min_, logarithmic transformation was applied before regression modelling. In the full-dataset univariate analysis, female gender, ventilator dependence ≥96 h, and CRRT were associated with higher log-transformed trough concentrations. Age, vancomycin dosing, PCT, Scr, SUN, and NT-proBNP also showed positive associations with log C_ss-min_ (Supplementary Table S2).

### Multivariable GEE for log-transformed C_ss-min_

Multivariable GEE with exchangeable working correlation structure was constructed to identify independent correlates of log C_ss-min_. Variables with P < 0.05 in univariate screening, plus clinically important covariates including IABP and body weight, were incorporated into the adjusted model. Model fit was evaluated using the quasi-likelihood under the independence model criterion (QICC).

In the primary model including 460 eligible TDM measurements, organ-support were independently associated with higher log-transformed trough concentrations: ventilator dependence ≥96 h (Exp(β) = 1.058, 95%CI: 1.015–1.102), CRRT (Exp(β) = 1.071, 95%CI: 1.020–1.125), and IABP (Exp(β) = 1.053, 95%CI: 1.004–1.103). Compared with the low-dose reference regimen (Group 1), higher dosing regimens were associated with elevated trough concentrations in a dose-dependent manner: Group 2 (0.5 g q8h, Exp(β) = 1.108, 95%CI: 1.050–1.169), Group 3 (0.5 g q6h, Exp(β) = 1.259, 95%CI: 1.182–1.342), and Group 4 (high-dose, Exp(β) = 1.240, 95%CI: 1.175–1.309). Positive independent associations were also observed for age (Exp(β) = 1.003, 95%CI: 1.002–1.005), Scr (Exp(β) = 1.001, 95%CI: 1.001–1.002), and SUN (Exp(β) = 1.007, 95%CI: 1.005–1.010), while body weight showed a negative association (Exp(β) = 0.998, 95%CI: 0.996–1.000). PCT, STB, and NT-proBNP were not statistically significant; operative duration reached statistical significance (p < 0.001) but with a negligible effect size (Exp(β) = 1.000), indicating no clinically meaningful association (Table 3).

**TABLE 3:** Primary multivariable generalized estimating equation (GEE) model for factors associated with log-transformed vancomycin steady-state trough concentration.

Parameters	β	SE	95% CI	Wald χ^2^	P-value	Exp(β)	95% CI for Exp(β)
Intercept	0.765	0.086	(0.597, 0.934)	79.193	<0.001	2.149	(1.816, 2.544)
Gender (female vs. male)	0.063	0.0232	(0.017, 0.108)	7.302	0.007	1.065	(1.017, 1.114)
Age	0.003	0.0009	(0.002, 0.005)	14.766	<0.001	1.003	(1.002, 1.005)
Ventilator dependence ≧96 h	0.056	0.021	(0.015, 0.097)	7.071	0.008	1.058	(1.015, 1.102)
CRRT	0.069	0.025	(0.020, 0.118)	7.636	0.006	1.071	(1.020, 1.125)
IABP	0.051	0.0239	(0.004, 0.098)	4.584	0.032	1.053	(1.004, 1.103)
Dosing regimen: Group 4 (high-dose regimen)	0.215	0.0276	(0.161, 0.269)	60.987	<0.001	1.240	(1.175, 1.309)
Dosing regimen: Group 3 (0.5 g q6h)	0.230	0.0324	(0.167, 0.294)	50.653	<0.001	1.259	(1.182, 1.342)
Dosing regimen: Group 2 (0.5 g q8h)	0.102	0.0274	(0.049, 0.156)	14.012	<0.001	1.108	(1.050, 1.169)
PCT	0.0002	0.0002	(-0.001, 0.0002)	0.804	0.37	1.000	(0.999, 1.000)
Scr	0.001	0.0002	(0.001, 0.002)	27.132	<0.001	1.001	(1.001, 1.002)
SUN	0.007	0.0013	(0.005, 0.010)	30.023	<0.001	1.007	(1.005, 1.010)
STB	−0.00005	0.0002	(-0.0005, 0.0004)	0.053	0.818	1.000	(1.000, 1.000)
Operative duration	−0.0000002	0.00000003	(−0.00000025, −0.00000014)	46.596	<0.001	1.000	(1.000, 1.000)
NT-proBNP	0.000002	0.000001	(−0.0000002, 0.0000037)	3.123	0.077	1.000	(1.000, 1.000)
Weight	−0.002	0.001	(-0.004, −0.0003)	4.949	0.026	0.998	(0.996, 1)

The dependent variable was log-transformed vancomycin steady-state trough concentration (logCss-min). IABP, was a pre--specified clinical hypothesis-driven covariate forced into multivariable GEE. A multivariable GEE, model with exchangeable working correlation structure was applied to account for repeated TDM, measurements within individual patients. Operative duration showed statistical significance but a negligible effect size (per-hour change), with no apparent clinical relevance. β = regression coefficient; SE, standard error; CI, confidence interval. Exp (B) represents the ratio of geometric mean Css-min relative to the reference group, derived from the log-transformed continuous outcome, and is not an odds ratio for binary events. Vancomycin dosing regimen was entered as a categorical covariate; Group 1 (low-dose regimen) served as the reference category and is not displayed in the table. Group 5 (non-standard regimen/temporary drug discontinuation, n = 13) was excluded from this multivariable model. All continuous correlates were entered in their original measurement units: age (years), weight (kg), operative duration (hours), PCT (ng/mL), Scr (μmol/L), SUN (mmol/L), STB (μmol/L), NT-proBNP (ng/L).

QICC, 43.954.

Sensitivity analysis restricted to 217 patients with at least one early-phase TDM included 365 eligible measurements after excluding non-standard regimens (Supplementary Table S4). Most correlates retained consistent direction and statistical significance compared with the primary model, including organ-support measures, dosing regimens, renal-related laboratory indices and body weight. Of note, age lost statistical significance in this subgroup sensitivity model (p = 0.095).

As a sensitivity analysis, we also re-ran the primary GEE model using continuous weight-adjusted daily dose of vancomycin variable; results were broadly consistent with the categorical-regimen specification (Supplementary Table S12), supporting the robustness of our main findings.

### Interaction sensitivity analyses

Interaction terms were further introduced to test for effect modification. In the 217-patient subgroup model incorporating predictor-by-postoperative-time interactions, only CRRT showed a statistically significant interaction with postoperative time period (p = 0.005), suggesting that the effect of CRRT on trough concentration varied between early and late postoperative phases. Ventilator dependence and IABP did not show significant time-dependent interactions (all p > 0.05) (Supplementary Table S5).

When testing dosing-regimen-by-organ-support interactions across the full 460-measurement dataset, no statistically significant interactions were detected. Only the interaction between dosing-regimen Group 2 and CRRT showed a borderline p-value of 0.087, which did not reach the predefined significance threshold (Supplementary Table S6).

### Logistic-GEE analyses for binary trough-concentration outcome

To further explore risk factors for supratherapeutic trough concentration (>20 mg/L), a multivariable logistic-GEE model was constructed with binary C_ss-min_ as the outcome, retaining original categorical covariates. Higher vancomycin dosing regimens were the strongest independent risk factors, with odds ratios increasing in a dose-dependent manner (Group 2: OR = 5.25; Group 3: OR = 20.85; Group 4: OR = 27.55; all p < 0.001). Female sex (OR = 3.13, 95%CI: 1.58–6.20, p = 0.001), elevated Scr (OR = 1.021, p < 0.001), and elevated SUN (OR = 1.104, p < 0.001) were also independently associated with supratherapeutic exposure (Supplementary Table S7).

A sensitivity logistic-GEE was additionally performed in which selected continuous covariates (age, Scr, SUN, STB) were dichotomized by clinical thresholds. The direction and significance of major risk factors remained largely consistent with the primary logistic-GEE model: higher dosing regimens, female sex, age >60 years, ventilator dependence ≥96 h, Scr above the upper limit of normal, and SUN above the upper limit of normal remained significant correlates of supratherapeutic trough concentration (Supplementary Table S8).

### Model discriminative performance

The discriminative performance of the logistic-GEE prediction model was evaluated using receiver-operating characteristic (ROC) analysis at the individual TDM-measurement level. The C-statistic (area under the curve, AUC) was 0.790 (95%CI: 0.748–0.832). The optimal probability threshold, determined by maximizing the Youden index, was 0.376, yielding a sensitivity of 78.1% and a specificity of 69.3% (Supplementary Table S9; Supplementary Figure 2). It should be noted that this ROC analysis treated each TDM measurement as an independent observation and did not adjust for within-patient correlation due to repeated sampling from the same individual, which represents a limitation of this discriminative performance assessment.

### Assessment of potential selection bias

Prespecified supplementary comparisons were conducted to evaluate selection bias related to postoperative sampling timing: comparison between early- and late-phase TDM observations (Supplementary Table S1), and baseline-characteristic contrast between patients with at least one early-phase TDM and those with exclusively late-phase TDM. Overall baseline characteristics were well balanced between the two groups, with the exception of ventilator dependence ≥96 h, which was significantly more prevalent among patients with exclusively late-phase TDM (53.0% vs. 32.3%, P = 0.002; Supplementary Table S10). Notably, prolonged ventilator dependence was also identified as a key correlate of higher vancomycin trough concentration in the primary multivariable GEE model, pointing to potential selection bias within this subgroup comparison.

Descriptive distributions of merged vancomycin dosing regimens are provided in Supplementary Table S11.

## Discussion

Patients undergoing CABG combined with concomitant valve surgery typically suffer from advanced cardiovascular diseases and comorbidities. Individualized vancomycin therapy in this specific subgroup is particularly challenging, primarily due to the high pharmacokinetic variability of vancomycin. Previous real-world studies of vancomycin have largely focused on pediatric and general critically-ill patients (Al-Sulaiti et al., 2025; Tsai et al., 2024; Sürmelioğlu et al., 2026; Wang et al., 2023; Vander Elst et al., 2026), while data specific to cardiac surgical patients remain limited. This study explored correlates of vancomycin trough concentrations in this real-world cardiac surgical cohort.

For patients with unstable renal function, therapeutic drug monitoring of vancomycin steady-state trough concentration and timely dose adjustment represents a practical clinical strategy compared with AUC_24_-guided dosing (He et al., 2020). Given repeated TDM measurements per patient, we applied generalized estimating equation (GEE) models to identify independent correlates of Css-min in patients recovering from combined CABG and valve surgery.

Our results showed that vancomycin exposure is shaped by a combination of traditional factors (age, dosing, renal function) and intensive-care supportive interventions, including IABP, CRRT, and prolonged mechanical ventilation. Vancomycin pharmacokinetics (PK) in this population are not merely driven by age and renal function but are also influenced by intensive-care support and dynamic postoperative clinical changes. Cardiac-surgery-related critical illness and organ-support therapies exert important influences on vancomycin exposure. Multiple studies have reported that CRRT may increase vancomycin clearance and predispose to sub-therapeutic trough concentrations, requiring dose escalation to achieve target exposure (Srour et al., 2023; Oda et al., 2020). In contrast, our study identified CRRT as an independent predictor of higher C_ss-min_.

Perioperative CRRT in cardiac surgery is commonly indicated for volume overload, severe acidosis, hyperkalemia, rhabdomyolysis, haemodynamic instability, and low-cardiac-output syndrome (Chinese Expert Consensus Working Group, 2019; Lameire and Kellum, 2013). Patients receiving CRRT frequently have cardiac insufficiency, disturbed fluid distribution, and fluctuating renal function. Both CRRT mode and intensity alter vancomycin PK, making PK prediction difficult (Wang et al., 2023). Cumulative vancomycin exposure and serum albumin have been reported as risk factors for supratherapeutic vancomycin concentrations among CRRT-treated patients (Liu et al., 2022). Inadequate dose adjustment according to CRRT settings may result in excessively high trough concentrations.

Prespecified interaction analyses revealed a significant CRRT-by-postoperative-time interaction (Supplementary Table S5), indicating that the impact of CRRT on vancomycin troughs differs across early and late postoperative periods. No significant time-dependent interactions were observed for mechanical ventilation or IABP. Moreover, we found no statistically significant dosing-regimen-by-organ-support interactions across the full dataset; only a borderline non-significant trend was observed for regimen Group 2 combined with CRRT, which should not be over-interpreted (Supplementary Table S6).

Vancomycin PK during CRRT are highly variable in critically-ill patients (Sürmelioğlu et al., 2026). Early-postoperative fluid shifts, capillary leak, and variable CRRT settings may jointly modify vancomycin distribution and elimination and contribute to this time-dependent effect. Therefore, TDM-guided individualized dosing, adjusted for CRRT mode, intensity and renal function, is essential to maintain optimal vancomycin exposure (Sürmelioğlu et al., 2026; Garreau et al., 2021; Smeets et al., 2023).

The effect of mechanical ventilation on vancomycin PK remains inconsistent across published critical-care studies (Medellín-Garibay et al., 2017; Smith et al., 2012; Perkins et al., 1989; Minkutė et al., 2013; Ghanbarveisi et al., 2026). Medellín-Garibay SE et al. reported mechanical ventilation could reduce vancomycin clearance by 20% in critically-ill patients (Medellín-Garibay et al., 2017). Perkins MW et al. suggested that mechanical ventilation itselt may not alter drug disposition; however, when combined with PEEP, it may impair renal perfusion and reduce clearance of GFR-dependent drugs such as vancomycin (Smith et al., 2012; Perkins et al., 1989).

Conversely, other investigators have linked mechanical ventilation to augmented renal clearance and lower vancomycin exposure (Minkutė et al., 2013). Whereas some authors observed no relevant PK differences between ventilated and non-ventilated patients (Ghanbarveisi et al., 2026).

In our cohort, prolonged mechanical ventilation ≥96 h was independently associated with higher vancomycin trough concentrations in both the full dataset and the early-phase subgroup. Such inter-study discrepancies likely originate from differences in patient case-mix, surgical procedures, ventilator parameters, and timing of TDM sampling. A key novel finding of the present study is that IABP support was consistently an independent predictor of elevated Css-min in both the overall dataset and the early-phase subgroup, an observation that has been sparsely described in prior cardiac-surgery vancomycin PK literature.

IABP is frequently deployed for refractory postoperative low-cardiac-output syndrome and cardiogenic shock after cardiac surgery (Werdan et al., 2014; Sharma et al., 2024). In such patients, impaired renal perfusion lowers GFR and reduces renal clearance of vancomycin, which is a hydrophilic drug (Cantu et al., 1994; He et al., 2021; Smith et al., 2012; Beach et al., 2017). Regional hypoperfusion may also limit drug transfer from plasma to peripheral tissues (Smith et al., 2012; Perkins et al., 1989; Joukhadar et al., 2001).

Therefore, higher trough concentrations among IABP-supported patients may partly stem from reduced renal vancomycin clearance secondary to underlying low cardiac output. IABP may act not only as a hemodynamic support device but also serve as a clinical marker for severe circulatory compromise, which alters vancomycin distribution and elimination (Werdan et al., 2014; Ley, 1993; Jiang et al., 2017). This finding carries clinical practical implications: clinicians should anticipate altered vancomycin PK and prioritize close TDM monitoring when managing patients receiving IABP support. Of note, IABP support did not emerge as an independent correlate of supra-therapeutic trough concentration C_ss-min_>20 mg/L in the binary logistic-GEE model. This suggests its association with higher vancomycin trough concentrations across the full concentration range does not necessarily translate into a clinically meaningful increase in the probability of crossing the nephrotoxicity-related threshold.

Consistent with prior reports (Rybak et al., 2009; Ding et al., 2026), Scr and SUN reflecting renal function showed positive independent correlations with Css-min in our full dataset. Age and vancomycin dosing regimen were also significant correlates, which aligns with classic pharmacokinetic expectations and reinforces their importance for dose estimation during TDM (Al-Sulaiti et al., 2025; Pongchaidecha et al., 2020).

In the sensitivity-analysis subgroup of 217 patients with at least one TDM measurement within postoperative day 7 (Supplementary Table S4), age lost statistical significance, whereas renal indices including Scr and SUN remained independent correlates of higher trough concentrations.

This observation regarding the loss of significance for age may reflect profound perioperative hemodynamic instability and rapidly fluctuating renal function after cardiac surgery. Even in this mixed-timing patient subgroup, serum-based renal biomarkers retained predictive value for vancomycin trough exposure (Smith et al., 2012).

ECMO and preoperative LVEF did not demonstrate significant associations in baseline stratified comparisons and univariate GEE screening; accordingly, these variables were not carried forward into the multivariable regression models. Although operative duration reached statistical significance in the primary model, its effect size was negligible with no obvious clinical relevance. PCT, STB and NT-proBNP showed no independent association with C_ss-min_ in the adjusted analysis.

### Clinical implications and future perspectives

Our findings support a dynamic, individualized approach to vancomycin TDM for patients undergoing combined CABG and valve surgery. Beyond conventional renal-function markers, clinicians should also consider intensive-care support measures including IABP, CRRT, and prolonged mechanical ventilation when interpreting vancomycin trough results and adjusting dosing. Given the significant CRRT-by-postoperative-time interaction identified in our analysis, special attention should be paid to changing postoperative clinical status, even among patients with stable serum creatinine. Frequent TDM and timely dose adjustment help accommodate fluctuating pharmacokinetics to reach target exposure and reduce nephrotoxicity risk (Reuter et al., 2022; He et al., 2020). This study work only identifies clinical correlates of C_ss-min_ and does not develop a ready-to-use dosing nomogram or prediction tool. These results may serve as preliminary evidence to inform future construction of individualized dosing strategies for this high-risk cardiac-surgical population.

### Limitations

This study has several inherent limitations associated with its observational retrospective design. First, data were collected from a single-center cohort, which may limit generalizability; and The present dataset mainly reflects retrospective pharmacokinetic surveillance rather than prospective, real-time dose adjustment guided immediately by trough concentrations. Some clinically relevant variables including fluid balance, lactate concentration, and vasoactive-agent dosage were not available for inclusion in the regression models. Second, our ROC-based discriminative performance assessment was computed at the TDM-measurement level and did not account for within-patient correlation caused by repeated sampling, which may bias model performance estimates. Third, the significant CRRT-by-postoperative-time interaction observed here requires external validation in independent cohorts. Future prospective multicenter studies are needed to validate these correlates and support the development of practical dosing nomograms or prediction tools for this cardiac-surgical population.

## Conclusion

Among patients undergoing combined CABG and concomitant valve surgery, vancomycin trough exposure is determined by both conventional correlates including age, dosing regimen and renal function, as well as intensive-care organ-support interventions such as IABP, CRRT, and prolonged mechanical ventilation. Notably, the effect of CRRT on vancomycin concentrations exhibits significant postoperative-time-dependent modification. These findings suggest that vancomycin dosing strategies in cardiac surgical ICU should move beyond reliance solely on renal-function indices. Clinicians are encouraged to adopt dynamic multi-factor assessment guided by TDM, adjusting dosing according to changes in hemodynamic support and postoperative clinical status to optimize vancomycin therapeutic exposure.

## Data Availability

The raw data supporting the conclusions of this article will be made available by the authors, without undue reservation.
